# Clinical profile of acute disseminated encephalomyelitis in children

**DOI:** 10.4103/1817-1745.76098

**Published:** 2010

**Authors:** M.P. Jayakrishnan, P. Krishnakumar

**Affiliations:** Department of Pediatrics, Institute of Maternal and Child Health, Medical College, Calicut, Kerala, India; 1 Institute of Mental Health and Neurosciences (IMHANS), Medical College, Calicut, Kerala, India

**Keywords:** Acute disseminated encephalomyelitis, Children

## Abstract

**Aim:**

To study the clinical profile of acute disseminated encephalomyelitis (ADEM) in children.

**Materials and Methods:**

All children admitted with ADEM during a period of one and a half years were included in the study. The diagnosis of ADEM was made based on the clinical presentation and suggestive MRI findings. All children were treated with intravenous methyl prednisolone, followed by oral prednisolone and followed up for varying periods up to three and a half years.

**Results:**

The sample consisted of 14 children with 11(79%) girls and 3 (21%) boys. The oldest child was 12 years and the youngest was a six-month-old infant. Acute febrile illness preceded the onset of neurological symptoms in 64% of children. The interval between the preceding illness and symptoms of ADEM varied from 7 days to 28 days (mean 12 days). The common presenting symptoms were fever, vomiting, headache, gait disturbance and generalized seizures. Neurological manifestations included altered sensorium, multiple cranial nerve involvement, quadriplegia and paraplegia, dystonia and choreiform movements, nystagmus, bladder involvement (both incontinence and retention), speech defect and double vision. Facial nerve was the most common cranial nerve involved. Psychological manifestations included aggressive behavior, psychotic symptoms and mood changes. One child each had features of acute psychotic episode and depressive episode. All children recovered fully. One child had multiphasic disseminated encephalomyelitis (MDEM) on follow up.

**Conclusion:**

Despite the serious neuropsychiatric manifestations, ADEM in children generally has good immediate outcome. Children with ADEM need long-term follow up for cognitive impairments.

## Introduction

Acute disseminated encephalomyelitis (ADEM) is a monophasic, immune-mediated demyelinating disorder of the central nervous system that can follow infections or immunizations.[1] The diagnosis of ADEM is based on the acute onset of neurologic signs and symptoms along with evidence of multifocal lesions of demyelination on neuroimaging.[1,2]

The present study aims to analyze the clinical profile of ADEM in children. The study was conducted at the Department of Pediatrics at Calicut Medical College. Calicut Medical College is a referral centre for the five northern districts of Kerala. The study includes data from May 2006 to December 2007.

## Materials and Methods

All children admitted to the Department of Pediatrics at the Institute of Maternal and Child Health, Medical College Calicut with the diagnosis of ADEM during the study period were included in the study. The diagnosis of ADEM was made based on the clinical presentation and suggestive MRI findings. At the time of admission, detailed history taking, clinical examination and relevant investigations were done and documented in the proforma. All children were treated with a standard protocol consisting of three day treatment with IV methylprednisolone 30 mg/kg followed by oral prednisolone 2 mg/kg for two weeks and tapered off within next two weeks along with symptomatic treatment. The children were followed up for varying periods up to three and a half years.

Statistical analysis was done by Epi info statistical package.

## Results

There were 14 children admitted with the diagnosis of ADEM during the study period. They included 11 (79%) girls and 3 (21%) boys. Three children were below the age of three years, 5 children in the 3-6 year age group and 6 children in the 6-12 year age group. The youngest was a six-month-old infant. All of them had first episode of the illness. There was no clustering of cases during any season.

Acute febrile illness preceded the onset of neurological symptoms in 9 (64%) children. Among them, 5 (36%) had upper respiratory tract infection and 4(27%) had non-specific fever. A two-year-old girl had diphtheria pertussis tetanus (DPT) immunization along with oral polio vaccine three weeks prior to the onset of symptoms. There was no child with history of exanthematous fevers or gastrointestinal illnesses prior to the onset of symptoms. No preceding illness could be identified in 5 (36%) children. The interval between the preceding illness and symptoms of ADEM varied from 7 days to 28 days (mean 12 days).

The common presenting symptoms were fever, vomiting, headache, gait disturbance and generalized seizures. Neurological manifestations included altered sensorium, multiple cranial nerve involvement, quadriplegia and paraplegia, dystonia and choreiform movements, nystagmus, bladder involvement (both incontinence and retention), speech defect and double vision. Facial nerve was the most common cranial nerve involved. Both LMN and UMN facial palsy occurred. Psychological manifestations included aggressive behavior, emotional lability, and irritable, elated or depressed mood. One child each had features of acute psychotic episode and depressive episode [Table 1]

**Table 1:** Clinical presentation of ADEM

Systemic symptoms	No.	Percentage
Fever at onset	9	64
Vomiting	6	43
Headache	4	29
Neurological manifestations		
Generalized seizures	3	21
Speech defect	4	29
Altered sensorium	3	21
Double vision	1	7
Aphasia	1	7
+Motor deficits	10	71
++Cranial nerve	5	36
Nystagmus	3	21
Abnormal movements	3	21
Urinary incontinence/ retention	3	21
Psychiatric manifestations		
Aggressive behavior	1	7
Acute psychosis	1	7
Mood changes	7	50

+ Quadriplegia (6), Paraplegia (4);

++ LMN-facial (3), UMN-facial (2), Abducent (1), palatal palsy (1)

The white blood cell count (WBC) ranged from 5400 to 15600/c mm with a mean of 10475 cells/c mm. Erythrocyte sedimentation rate (ESR) ranged from 5 to 70 mm/h with a mean of 43 mm/h. None of the children had cerebrospinal fluid (CSF) pleocytosis. Mean CSF protein was 42 mg% with a range of 11 mg% to 92 mg%. CSF sugar was normal in all cases and oligoclonal bands in the CSF could not be done in any of the cases due to lack of facilities.

### Magnetic resonance imaging findings

Magnetic resonance imaging (MRI) was done in all children. The area involved in the majority of children was the parietal lobe. Lesions were noted in the subcortical white matter, mid brain, pons, corpus callosum, basal ganglia, medulla and cerebellum. One third of children had spinal cord involvement [Table 2].

**Table 2:** Site of lesion in MR imaging

Location of hyperintense lesions on T2/FLAIR images	No. *N*=14	Percentage
Frontal lobe	5	36
Parietal lobe	8	57
Temporal lobe	4	29
Occipital lobe	2	14
Cortical grey matter	2	14
Subcortical white matter	6	43
Periventricular white matter	1	7
Internal capsule	1	7
Thalamus	1	7
Basal ganglia	3	21
Pons	4	29
Mid brain	4	29
Medulla	1	7
Corpus callosum	4	29
Cerebellum	1	7
Cerebellar peduncle	2	14
Cervical cord	3	21
Thoracic cord	5	36
Lumbosacral	1	7
Centrum semiovale	2	14
Corona radiata	2	14
Peritrigonal area	1	7

### Follow up MRI

Follow- up MRI was performed in six children. Among them, five children had follow up MRI at three months and one had repeat MRI at five months after discharge from the hospital. All of them except one child had complete resolution of the lesions in the follow up MRI.

### Outcome

10(71%) children had total remission of symptoms within one week of starting steroids. 4(29%) children had residual symptoms at the end of steroid therapy. One child who presented with multiple cranial nerve involvement had persistent palatal palsy at the time of stopping steroids. A seven year old girl who presented with aphasia, altered sensorium and extrapyramidal involvement developed generalized seizures while on steroids and subsequently developed behavior disorder characterized by aggression. She also had persistent nominal aphasia. This child was treated with carbamazepine and risperidone in addition to steroids. The child who presented with acute psychotic episode had persistent behavior problems even after six weeks of steroid therapy and the repeat MRI after three months showed persistence of lesions. In this child, there was family history of mental illness and risperidone had to be continued for several months. A four-year-old child who presented with paraplegia had mild residual weakness of left lower limb at the time of stopping steroids.

### Long-term follow up

The children were followed up for a variable period from two months to three and a half years. Most of the cases made uneventful recovery.

One girl, who had recovered fully, died of unrelated illness after about six months of discharge from the hospital.

An eight-year-old boy, who was initially admitted with altered sensorium, multiple cranial nerve palsy and cerebellar signs, recovered fully after treatment and MRI at three months was normal. After an asymptomatic period of seven months, he presented with seizures and left hemiplegia. He had a nonspecific URI, two weeks prior to the illness. His MRI this time showed fresh lesions at a different location. He recovered fully and three years after the initial diagnosis, he continues to be asymptomatic with normal school performance.

Intelligence assessment could be done in eight children. Among them, 4 (50%) children had borderline IQ and 4 (50%) had normal IQ.

## Discussion

In the present sample, ADEM was found to occur in all age groups of children. The youngest was a six-month-old infant and the oldest child was 12-years-old. Studies in the past have reported comparable findings.[3–7]

Girls constituted 79% of children in the present sample. Studies from India and abroad have reported that ADEM is more common in boys,[2–4,8,9] while multiple sclerosis, the other demyelinating disorder with many similarities, is more common in women.[10,11] The reason for this female preponderance in the present sample should be evaluated in view of the disproportionate increase in the incidence of multiple sclerosis in women in recent studies.[12,13]

In the present sample, cases occurred throughout the year without any seasonal preference, in contrast to western data where predominance during winter and spring is reported[2,8]

The majority of children in the present sample had a nonspecific febrile illness preceding the onset. No child had preceding viral exanthema, a finding consistent with many of the studies in the developed world.[2,3,6,8,14] Indian studies have shown increased incidence of specific viral infections like measles, rubella and mumps.[15,16] This is expected in a state like Kerala, where most of the children are immunized and the incidence of vaccine preventable diseases has come down. The average duration between the preceding illness and ADEM was 12 days. This is consistent with the findings of other studies.[2,8,16]

The clinical features of ADEM in the present sample were comparable to those of previous reports.[2,4,5,8,16] One difference noticed was the absence of optic neuritis in the present sample. Optic neuritis is reported to occur in 3-35% of cases in various reports.[7,8] Also, CSF pleocytosis which is described in 28-65% of cases of ADEM[2,8,16,17] was not present in the present sample.

The child who had recurrence of neurological symptoms after a period of seven months could be considered as multiphasic acute disseminated encephalomyelitis (MDEM) as per the definition.[17]

On follow up, it was found that no child had residual neurological deficits. Among those in whom intelligence could be assessed, 50% had borderline intelligence. It has been reported that in spite of the absence of gross neurological deficits, incidence of behavior and emotional problems are higher in children with ADEM. Children in whom the disease occurred before the age of five years were found to be more vulnerable to develop emotional and behavioral problems. Hence, there is a need for long-term follow up of children with ADEM.[18]

## Conclusion

Despite the serious neuropsychiatric manifestations, ADEM in children generally has a good outcome. Children with ADEM need long-term follow up for cognitive impairments and emotional problems. Clinical presentation of ADEM in the present sample is comparable to previous studies except for the female preponderance. Further studies are required to analyze the reason for this female preponderance.
